# The Genomic 3′ UTR of Flaviviruses Is a Translation Initiation Enhancer

**DOI:** 10.3390/ijms23158604

**Published:** 2022-08-03

**Authors:** Alfredo Berzal-Herranz, Beatriz Berzal-Herranz, Sara Esther Ramos-Lorente, Cristina Romero-López

**Affiliations:** Instituto de Parasitología y Biomedicina “López-Neyra” (IPBLN), CSIC, PTS Granada, Avenida del Conocimiento 17, 18016 Armilla, Granada, Spain; beatriz.berzalh@ipb.csic.es (B.B.-H.); seramos@ipb.csic.es (S.E.R.-L.)

**Keywords:** West Nile Virus, WNV, flavivirus, regulation of translation, 3′ UTR, cap-independent translation

## Abstract

Viruses rely on the cellular machinery of host cells to synthesize their proteins, and have developed different mechanisms enabling them to compete with cellular mRNAs for access to it. The genus *Flavivirus* is a large group of positive, single-stranded RNA viruses that includes several important human pathogens, such as West Nile, Dengue and Zika virus. The genome of flaviviruses bears a type 1 cap structure at its 5′ end, needed for the main translation initiation mechanism. Several members of the genus also use a cap-independent translation mechanism. The present work provides evidence that the WNV 5′ end also promotes a cap-independent translation initiation mechanism in mammalian and insect cells, reinforcing the hypothesis that this might be a general strategy of flaviviruses. In agreement with previous reports, we show that this mechanism depends on the presence of the viral genomic 3′ UTR. The results also show that the 3′ UTR of the WNV genome enhances translation of the cap-dependent mechanism. Interestingly, WNV 3′ UTR can be replaced by the 3′ UTR of other flaviviruses and the translation enhancing effect is maintained, suggesting a molecular mechanism that does not involve direct RNA-RNA interactions to be at work. In addition, the deletion of specific structural elements of the WNV 3′ UTR leads to increased cap-dependent and cap-independent translation. These findings suggest the 3′ UTR to be involved in a fine-tuned translation regulation mechanism.

## 1. Introduction

The regulation of translation is probably the main process controlling gene expression in positive, single-stranded RNA viruses. Like any other virus, RNA viruses possess no translation machinery of their own. Viral translation thus relies on the usurpation of the host cell’s machinery, requiring infecting virus particles use molecular mechanisms that allow them to compete efficiently with cellular mRNAs. Like many other viruses, and most cellular mRNAs, the members of the genus *Flavivirus* use the cap-dependent translation initiation mechanism [1,2]. The cap (5′-m^7^GpppN) is required for recruiting the eukaryotic translation initiation factor complex eIF4F, a pre-requisite for binding to the 40S ribosomal subunit and subsequent scanning for an appropriate translation initiation codon [3]. In addition to this canonical mechanism, RNA viruses use different cap-independent mechanisms to gain an advantage over cellular mRNAs in the initiation of translation [4]. Several viruses, including some flaviviruses, are reported to make use of more than one translation initiation mechanism to ensure their fitness [5,6,7].

The genus *Flavivirus* has many members, including numerous human pathogens. Their genome consists of a single-stranded RNA molecule about 11,000 nt long that bears a single open reading frame (ORF) flanked by untranslated regions (UTRs). These UTRs vary in length from one type of flavivirus to another, but all are composed of universally conserved structural elements with essential functional roles in the viral cycle (for a review see [8,9,10]). The genome possesses a type I cap structure at the 5′ terminus and lacks a polyA tail at the 3′ end [11,12]. This determines that these viruses have a major translation initiation mechanism in which the functions of the polyA tail are achieved via the highly conserved structural elements of the 3′ UTR [10,13,14,15]. Edgil et al. showed that Dengue virus (DENV) RNA can also be translated under conditions in which cap-dependent translation initiation is arrested [5], switching between cap-dependent and cap-independent mechanisms of translation initiation to efficiently compete for the host cell translation machinery. This was later confirmed by Song et al. who also showed the same to be true for the closely related Zika virus (ZIKV) [6]. More recently it has been shown that the cap-independent initiation mechanism is also used by three other flaviviruses: Duck Tembusu virus (DTMUV), Tembusu virus (TMUV), and Japanese Encephalitis virus (JEV) [7]. There is no consensus on how the cap-independent translation mechanism works, and the existence of IRES-activity (internal ribosome entry site) at the 5′ UTR is controversial, but all reports agree on the involvement of the 3′ UTR. It has previously been reported that some RNA viruses use translation initiation mechanisms different from both the IRES-dependent and cap-dependent types, but rather depend on cis-acting enhancers of translation (CITEs) that promote 5′ end initiation [16,17,18]. Furthermore, many plant RNA viruses use cis-acting enhancers of translation located in their 3′ UTRs (known as 3′ CITEs). These are characterized by well-defined secondary structures that allow the recruitment of translation initiation factors [19,20].

The present work provides evidence that West Nile Virus (WNV), a well-studied mosquito-borne flavivirus, also uses a 5′ cap-independent translation initiation mechanism which is dependent on the 3′ UTR of the viral genome. This further supports the idea that this might be a mechanism used by all flaviviruses. Interestingly, the WNV 3′ UTR can be substituted by the genomic 3′ UTR of other flaviviruses and cap-independent translation initiated, suggesting that the molecular mechanism behind the enhancement function of the genomic 3′ UTR involves no direct interaction between the two ends of the viral genome. Evidence that the 3′ UTR of flaviviruses functions as a general enhancer of translation is also provided.

## 2. Results

### 2.1. Cap-Independent Initiation of Translation

**Figure 1:**
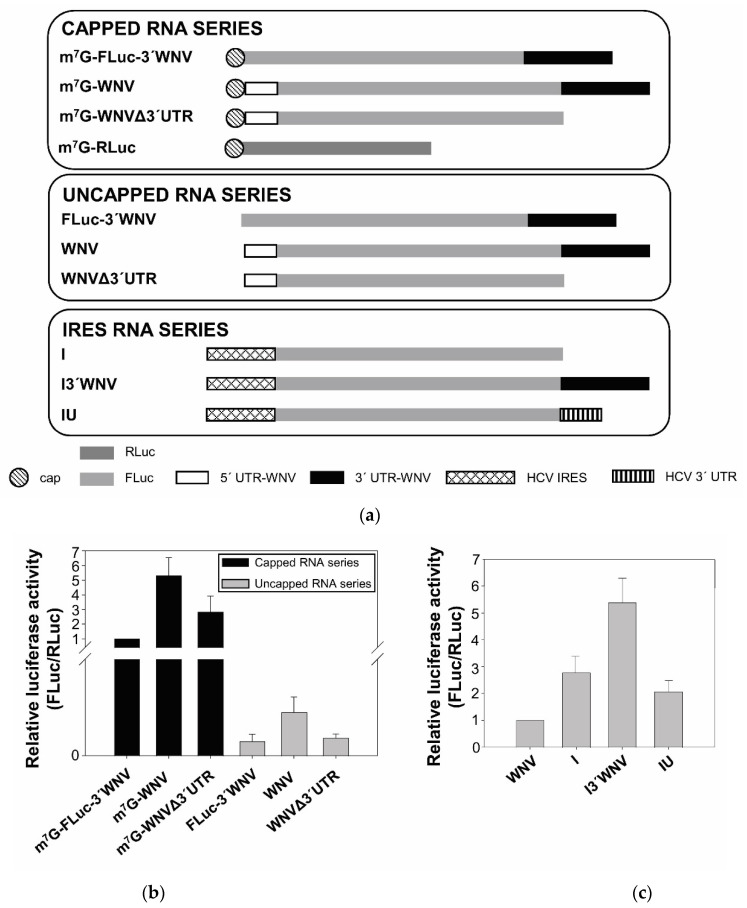
Cap-independent translation of WNV genome. (**a**) Diagram of the RNA constructs harbouring the FLuc coding sequence flanked by different 5′ end and the 3′ UTR of the WNV RNA genome, and control constructs. (**b**) Translation efficiency of different capped and uncapped RNA constructs. Vero cells were cotransfected with the individual RNA constructs indicated in (**a**) and m^7^G-RLuc RNA. Translation activity was determined as FLuc protein activity normalized against the value obtained for RLuc. Values refer to that of the capped RNA coding the FLuc fusion to the WNV 3′ UTR (m^7^G-FLuc-3′UTR). (**c**) Translation efficiency of the HCV IRES-dependent RNA constructs. Translation activity was determined as described in (**b**); values are referred to that of the uncapped WNV construct. Values are the mean of at least three independent experiments ± standard deviation.

To explore the idea that cap-independent translation might be a general strategy followed by flaviviruses, the ability of the 5′ end of WNV to initiate translation was tested in the absence of the 5′ cap. For this, a series of subgenomic viral RNAs was constructed. The series-head RNA construct, named m^7^G-WNV, consisted of a 5′ capped RNA containing the coding sequence of firefly luciferase (FLuc) flanked by the 5′ end (nt positions 1–160) and the complete 3′ UTR of the NY99-flamingo382-99 WNV strain (nt positions 10,399 to 11,029). The m^7^G-WNVΔ3′UTR RNA lacked the WNV 3′ UTR (Figure 1a). Both RNAs were also synthesized in the absence of the 5′ cap, yielding the WNV and WNVΔ3′UTR constructs, respectively. A control construct, m^7^G-FLuc-3′WNV, lacking the WNV 5′ end fragment along with the corresponding uncapped RNA construct (FLuc-3′WNV), was also synthesized. The translation efficiency of each of these constructs was measured in Vero cells. Cells were cotransfected with each individual RNA construct and cap-dependent RLuc mRNA (m^7^G-RLuc) to normalize transfection efficiency.

All the capped RNAs were efficiently translated, although m^7^G-WNV did so five times as efficiently as the control m^7^G-FLuc-3′WNV (Figure 1b). The translation of the uncapped RNAs was very inefficient—at least 100 times lower than the corresponding capped RNAs. Interestingly, the uncapped WNV construct was repeatedly translated approximately 2.5 times as efficiently as the uncapped RNAs FLuc-3′WNV and WNVΔ3′UTR. These results suggest the existence of a cap-independent translation mechanism occurring at the WNV 5′ UTR, in good agreement with observations made for other flaviviruses [5,6,7].

Importantly, they also indicate this mechanism to be dependent on the genomic WNV 3′ UTR. In the presence of the 3′ UTR, translation increased by a factor of 2–2.5 in both in the presence and absence of the cap (compare m^7^G-WNV with m^7^G-WNVΔ3′UTR and the corresponding uncapped RNAs; Figure 1b).

Since the cap-independent translation observed for the 5′ end of the WNV seems to be less efficient than that observed for other flaviviruses, its activity was compared with the less efficient hepatitis C virus (HCV) IRES (Figure 1c) in Vero cells. The translation of the HCV IRES (construct I in Figure 1c) was triple that of the uncapped WNV. Interestingly, the addition of the WNV 3′ UTR (I3′WNV) doubled the translation efficiency of the HCV IRES, whereas no increase was observed after the addition of the 3′ end of the HCV virus (IU). These results confirm the enhancing role of the WNV 3′ UTR in cap-independent translation, and do not support the existence of an IRES-dependent translation mechanism in WNV (at least of similar strength to those known).

**Figure 2:**
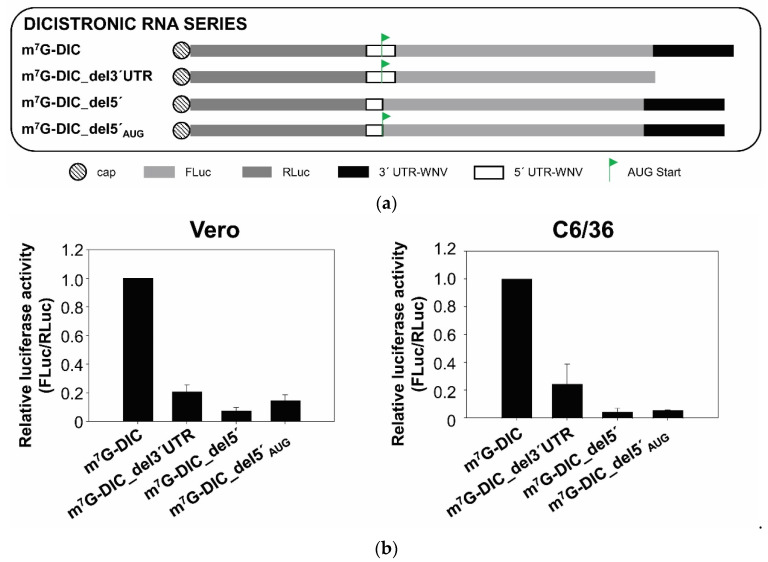
The 5′ end of the WNV genome promotes the internal initiation of translation. (**a**) Diagram of the dicistronic RNA constructs harbouring the capped RLuc mRNA at the 5′ end of the FLuc coding sequence flanked by the 5′ end, or truncated variants (del and del_AUG_, respectively) and the 3′ UTR of the WNV RNA genome. (**b**) Translation efficiency of dicistronic RNA constructs. Vero and C6/36 cells were transfected with an RNA construct indicated in (**a**); left and right panels respectively). Translation activity was determined as the FLuc protein activity and normalised against the value obtained for RLuc. Values refer to that of the dicistronic RNA containing the 5′ end (nt 1–160) of the WNV m^7^G-DIC genome.

To further examine the putative existence of an IRES at the 5′ end of the WNV RNA genome, a series of dicistronic constructs were assayed (Figure 2): (i) RNA m^7^G-DIC, which carries a first ORF encoding the reporter RLuc protein (the synthesis of which is cap-dependent) followed by the cassette including the 5′ UTR of WNV directing the synthesis of the FLuc protein, ending with the WNV 3′ UTR; (ii) the RNA m^7^G-DIC_del3′UTR, which lacks the WNV 3′ UTR; (iii) the RNA m^7^G-DIC_del5′, which only bears nt 1–95 of the 5′ UTR, and lacks the AUG start codon; and (iv) the RNA m^7^G-DIC_del5′_AUG_, which lacks the 3′ end of the 5′ UTR but retains the AUG codon at position 96. The AUG of the fluc gene was deleted in all these constructs in order to examine the role of the 5′ UTR in cap-independent translation.

These RNAs were used to transfect both Vero and C6/36 cells. At 4 h post-transfection, both cell lines showed slight but significant FLuc activity resembling that observed in assays with monocistronic constructs lacking the cap, thus demonstrating that cap-independent internal translation initiation occurs at the 5′ UTR of WNV (if only to a small extent). Importantly, this translational activity was extremely dependent on the presence of the 3′ UTR (Figure 2b), as demonstrated for the monocistronic constructs (see Figure 1). The assay with m^7^G-DIC_del5′_AUG_ also revealed that the domains located downstream of the WNV AUG start codon are critical for preserving cap-independent protein synthesis, which is in good agreement with previous results indicating the importance of the cHP domain for ribosomal positioning [21].

Taken together, these results highlight the critical role of the 3′ UTR as a translation enhancer in WNV, and reveal the existence of a minor, but detectable, internal translation initiation that supports cap-independent translational activity at the WNV 5′ end, which is dependent on the 3′ UTR.

### 2.2. The WNV 3′ UTR Enhances Translation in Mammalian Cells

**Figure 3:**
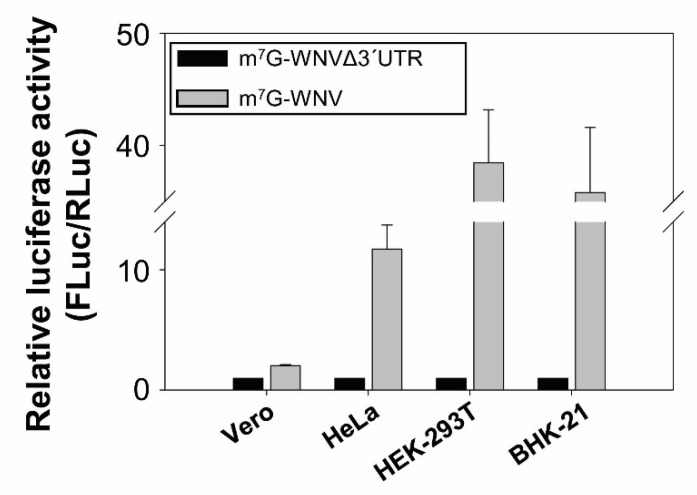
Translation enhancing effect of the 3′ UTR of the WNV genome in different mammalian cell lines. Different cell lines were cotransfected with the capped or uncapped RNA constructs harbouring the 5′ end of the WNV RNA genome fusion at the 5′ end of the FLuc RNA sequence followed, or not, by the WNV 3′ UTR and m^7^G-RLuc RNA. Translation activity was determined as described in Figure 1. Values refer to that of the corresponding construct lacking the WNV 3′ UTR, and are the mean of three independent experiments ± standard deviation.

To check whether the translation enhancer activity of the WNV 3′ UTR occurred in cells other than Vero cells, Hela, HEK-293T and BHK-21 cells were cotransfected with m^7^G-WNV, m^7^G-WNVΔ3′UTR or m^7^G-RLuc. In all cell lines, the presence of the genomic WNV 3′ UTR resulted in a significant enhancement of 5′ end translation initiation (Figure 3), with an increase by a factor of 10 in HeLa cells, of 20 in BHK-21 cells, and of 50 in the HEK-293T cells. Unexpectedly, in all these cell lines, the translation enhancing effect of the WNV 3′ UTR was greater than in Vero cells (only double).

**Figure 4:**
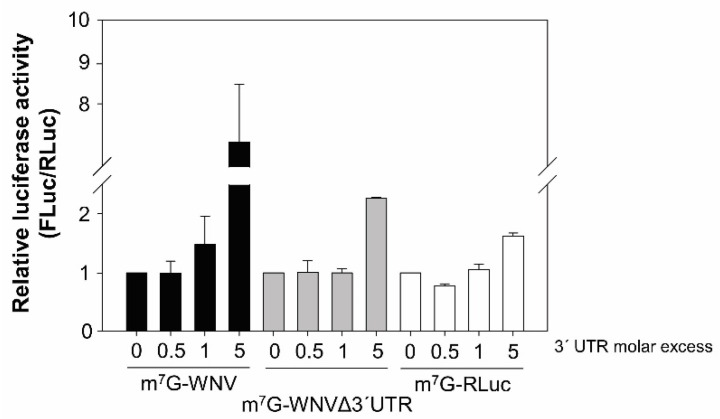
The genomic 3′ UTR of WNV enhanced translation in trans. Vero cells were cotransfected with the m^7^G-WNV or m^7^G-WNVΔ3′UTR RNA constructs, and m^7^G-RLuc RNA, in the presence of increasing molar excess of the genomic 3′ UTR WNV RNA fragment and decreasing amounts of the RNA-100 to compensate for the total RNA load in the transfection mix. Translation activity was determined as described in Figure 1. Values refer to that of the corresponding experiment without the 3′ UTR RNA fragment added.

To further investigate the translation enhancement exerted by the genomic 3′ UTR, tests were performed to see whether this activity was maintained in trans. Vero cells were cotransfected with m^7^G-WNV or m^7^G-WNVΔ3′UTR and increasing amounts of an RNA fragment formed only by the WNV genomic 3′ UTR. The cotransfection mix was complemented with the non-related RNA-100 to preserve the total amount of RNA [22]. The 3′ UTR provided in trans at a molar ratio of 1:5 increased the translation of both RNA constructs, although this was more notable for the construct containing the 3′ UTR (by a factor of seven versus 2.5) (Figure 4). Surprisingly, a slight enhancement of translation was also observed for the m^7^G-RLuc at the same molar ratio (1:5) (Figure 4). These results are in good agreement with those reported above that suggest that the WNV 3′ UTR shows a general translation enhancing activity.

### 2.3. The Genomic 3′ UTRs of DENV and YFV Also Function as Enhancers of 5′ WNV Translation

**Figure 5:**
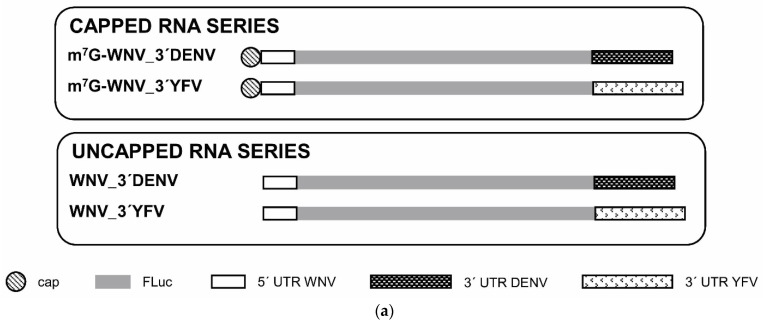

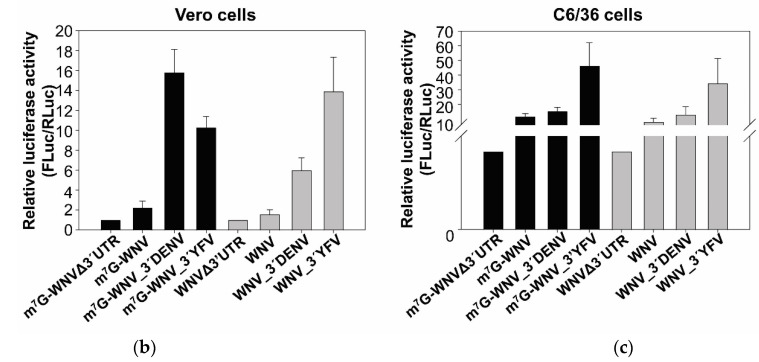
WNV translation initiation is enhanced by the 3′ UTRs of DENV and YFV. (**a**) Diagram of the chimeric RNA constructs harbouring the FLuc coding sequence flanked by the 5′ end of the WNV genome and the 3′ UTR of the DENV or YFV RNA genome. Translation efficiency of different capped and uncapped RNA constructs was determined in Vero and C6/36 cell lines. (**b**) Vero cells were cotransfected with each individual chimeric RNA construct and m^7^G-RLuc RNA. Translation activity was determined as described in Figure 1. Values are referred to that of the corresponding construct lacking any 3′ UTR fragment. (**c**) Mosquito C6/36 cells were cotransfected as in (**b**) and translation activity determined as described in Figure 1. Values are the mean of three independent experiments ± standard deviation. Black and grey bars correspond to the values of the capped and uncapped RNA constructs, respectively.

In an attempt to determine the molecular mechanism involving the 3′ UTR of WNV in enhancing translation at the genomic 5′ end, a series of chimeric RNA constructs were obtained with the genomic 3′ UTRs of DENV or YFV instead of the 3′ end of WNV (m^7^G-WNV-3′DENV and m^7^G-WNV-3′YFV, respectively; Figure 5a).

Vero cells were cotransfected with each individual RNA and the m^7^G-RLuc. The two heterologous 3′ UTRs acted as translation enhancers of both capped and uncapped WNV RNAs (Figure 5b). Interestingly, while the DENV 3′ UTR induced the greatest translation activity among the capped RNA series (by a factor of 16 for m^7^G-WNVΔ3′UTR, versus 4 and 11 for the 3′ UTRs of WNV and YFV, respectively), the 3′ UTR of YFV was the strongest enhancer of the corresponding uncapped RNA (WNVΔ3′UTR; Figure 5b)—by a factor of 14, versus 2 and 6 for the 3′ UTRs of WNV and DENV, respectively. These results suggest that the genomic 3′ UTRs of flaviviruses function as enhancers of translation. They also confirm that the 5′ end of the WNV genome uses a cap-independent translation mechanism, the activity of which is dependent on flavivirus genomic 3′ UTR. In other words, this dependence on the 3′ UTR does not necessarily mean that it has to be the 3′ UTR of the same virus species.

A parallel analysis performed in C6/36 mosquito cells showed qualitatively similar results to those obtained in mammalian cells. In this cellular background, a cap-independent translation mechanism at the 5′ end of WNV end was also observed (Figure 5c). Furthermore, the genomic 3′ UTR of the three flavivirus (WNV, DENV and YFV) promoted an increase in the translation activity of the WNV 5′ end, with the 3′ UTR of YFV performing better than DENV, and the latter better than WNV (Figure 5c), both for capped and uncapped RNAs. Interestingly, the magnitude of translation enhancement was significantly greater in this cell line than in Vero cells (compare Figure 5b,c). The 3′ UTR of WNV led to increases by factors of approximately 11 and 8 over the corresponding capped and uncapped RNA, respectively. The 3′ UTRs of DENV and YFV led to translation increases by factors of 15 and 46 for the capped RNAs, and by 13 and 34 for the uncapped RNAs, respectively.

### 2.4. The SL-III Structural Element of the WNV 3′ UTR Plays an Important Role in Regulating the Enhancement of Viral Translation

**Figure 6:**
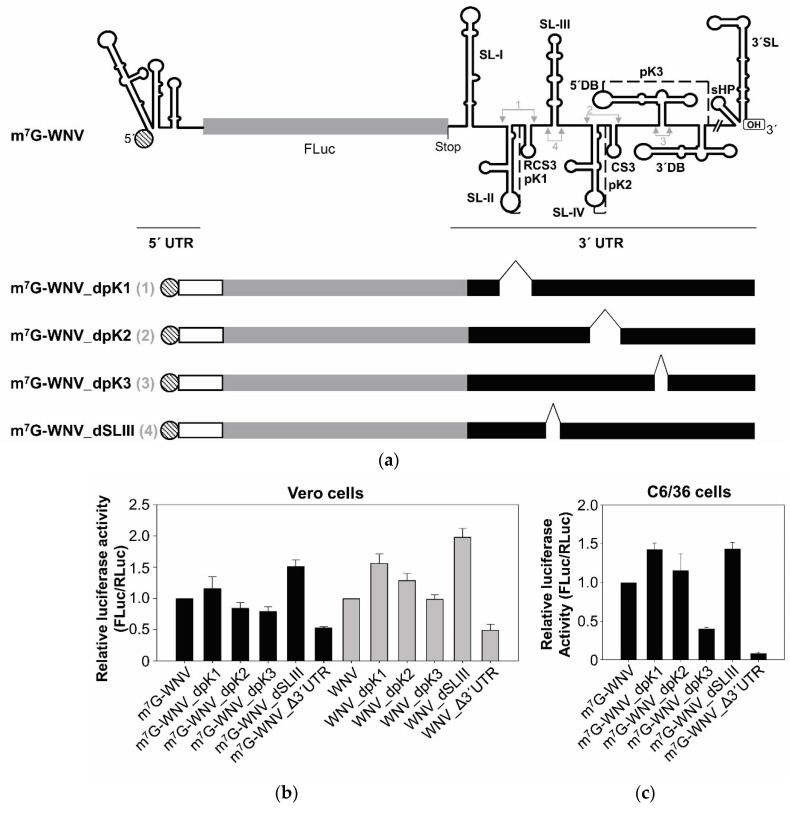
Highly conserved structural elements of the WNV 3′ UTR contribute to the regulation of 5′ end translation initiation. (**a**) Secondary structure of the WNV subgenomic RNA construct; the secondary structure elements of the 5′ and 3′ ends of the WNV genome are represented. Arrows indicate the first and last nucleotide included in the deletions to generate the 3′ UTR variants dPK1, dPK2, dPK3 and dSL-III (1–4, respectively). A diagram of the deletion RNA construct variants is shown in the lower panel. The key for the different bars is as in Figure 1. (**b**) The translation activity of different capped and uncapped RNA constructs carrying variants of the WNV 3′ UTR was determined in Vero cells. Cells were cotransfected with each individual 3′ UTR variant RNA construct and m^7^G-RLuc RNA. Translation activity was determined as described in Figure 1. Values refer to that of the corresponding wild type 3′ UTR construct. (**c**) The translation activity of different capped RNA constructs carrying variants of the WNV 3′ UTR was determined in C6/36 cells. Mosquito cells were cotransfected as in (**b**) and translation activity determined as described in Figure 1. Values are the mean of three independent experiments ± standard deviation. Black and grey bars correspond to the values of the capped and uncapped RNA constructs, respectively.

The genomic 3′ UTR of flaviviruses are rich in secondary structural elements that are highly conserved across different viral isolates [9,10] (Figure 6a). Many different reports have described their functional implications in the viral cycle [8,9,10,23,24]. Among them, pseudoknots, which have been shown to play essential roles in the WNV cycle, have been reported in other viruses to be involved in the regulation of translation [25,26,27]. A series of WNV 3′ UTR deletion mutants was thus obtained to examine the potential of the pseudoknot structures (PK1; PK2 and PK3; Figure 6a). A mutant lacking the SL-III element (located in-between the structural elements involved in the formation of the PK1 and PK2) was also tested (Figure 6a). The resulting constructs were named dPK1, dPK2, dPK3 and dSLIII, respectively. Vero cells were cotransfected with each individual capped or uncapped RNA and m^7^G-RLuc. All of the tested mutants allowed for greater translation efficiency than did the construct lacking the entire WNV 3′ UTR (WNVΔ3′UTR; Figure 6b).

For the capped RNAs, the deletion of the SL-III resulted in the highest increase (triple) with respect to the control m^7^G-WNVΔ3′UTR. Interestingly, the translation activity of the dSLIII construct was even greater than that seen with the m^7^G-WNV construct which carries the complete 3′ UTR (double). A slight increase (compared to the complete 3′ UTR) was also seen for the construct lacking the PK1 element. In the absence of the 5′ cap, the translation enhancing effect was greater still for all the mutants; all were associated with greater translation efficiency than the WNV construct—by a factor of 4 for the dSLIII mutant (Figure 6b). The effect of the complete 3′ UTR was maintained at a factor of around 2. Despite the existence of specific structural elements within the 3′ UTR that have a negative effect on translation, e.g., SL-III, the entire 3′ UTR had an enhancing effect.

The series of mutant capped constructs was also tested in mosquito C6/36 cells, and similar results were returned (Figure 6c). The deletion of the SL-III domain yielded an increase in translation similar to that seen in the Vero cells. In this new cellular context, dPK1 and dPK2 constructs also showed higher translation activities than the control m^7^G-WNV, with that for dPK1 returning values similar to those recorded for dSLIII, while the dPK3 construct was associated with less translation activity than m^7^G-WNV—which is in good agreement with the data reported for 3′ UTR deletion mutants of DTMUV [7].

## 3. Discussion

The observation that translation of the DENV genome is resistant to cap-dependent translation arrest conditions led to the hypothesis of the existence of a general cap-independent translation mechanism in flaviviruses [5]. Indeed, data supporting this hypothesis have been reported in at least five additional flaviviruses [6,7]. The present results are also consistent with previous reports suggesting the WNV genome to use a cap-independent translation initiation mechanism (Figure 1 and Figure 2). There is currently no consensus regarding whether the molecular mechanism underlying this is IRES-dependent or not, and the arguments for both seem to be based on the magnitude of the observed cap-independent translation activity of the studied genomic 5′ end compared to that associated with well-known IRESs.

The present results show that the WNV cap-independent translation mechanism works both in mammalian and insect cells (Figure 1, Figure 2 and Figure 5), although not with any great efficiency under the used experimental conditions. They also indicate the existence of an inefficient but significant internal translation initiation mechanism dependent on the genomic 3′ UTR, suggesting that an IRES-like region exists in the 5′ UTR of the WNV genome (Figure 2). It is tempting to propose that this mechanism operates best under certain environmental conditions, such as stress, in which cap-dependent translation might be abolished. Certainly, this kind of IRES is found not only in viruses but in many cellular mRNAs that make use of their 5′ UTRs in alternative translational mechanisms when the canonical translation pathways are blocked (for a review, see [28]). This translation mechanism used by flaviviruses might also be shared by those cellular mRNAs whose translation is increased under stress conditions, when the canonical translation is arrested, as happens during flavivirus infection [29].

The present results also provide evidence that cap-independent translation initiation depends on the presence of the genomic 3′ UTR (Figure 1 and Figure 2); this agrees with that reported for other flaviviruses [5,6,7]. The molecular mechanism underlying this dependence on the 3′ UTR remains unknown. It has been ruled out, in other flaviviruses, that the 3′ UTR effect is due to differences in RNA stability [5,6,7]. Enhancement of translation by the 3′ UTR has also been described in HCV, another member of the family *Flaviviridae* [30,31,32], for which we have previously shown that complete deletion of the HCV 3′ UTR does not result in changes in RNA stability that could explain the differences in translation efficiency that it causes [31]. Although we cannot rule out that changes in the RNA stability resulting from deletion of the WNV 3′ UTR may be responsible, at least in part, for the translation reduction observed in the present work, the enhancement of translation by the 3′ UTR in trans supports a specific effect. This would be in line with the previous report, where differences in RNA stability between DENV-based monocistronic constructs bearing different 3′ ends [3′ UTR of DENV, poly (A), minus poly(A)] were discarded as responsible for variations in their translational efficiency [6]. Interestingly, in the present work, the genomic 3′ UTR of WNV also enhanced cap-dependent translation initiation at the genomic 5′ end. A defining feature of flavivirus genomes is the existence of complementary sequence motifs at both genome ends—which are involved in genome cyclization [10,33]. It is thus tempting to think that the already proven direct RNA/RNA interactions between such motifs, which have been shown to be involved in replication [34,35,36,37,38,39], might also be involved in the translation-enhancing effect mediated by the 3′ UTR. However, the genomic cyclization mediated by these RNA/RNA interactions inhibits the initiation of translation in ZIKV and DENV [40]. In addition, the surprising finding that this enhancing effect is even greater in the presence of the genomic 3′ UTR of other flavivirus (Figure 5) argues against a mechanism involving a direct RNA/RNA interaction through sequence complementarity. Thus, the molecular mechanism underlying translation enhancement most likely involves the recruitment of host factors involved in the regulation of translation. It cannot be ruled out, however, that the 3′ UTR promotes the structural reorganization of the 5′ end through long-range tertiary interactions. In this respect, it has been shown by SHAPE and HMX analysis that the 5′ and 3′ UTRs of the genome of HCV mutually induce the reorganization of their overall structure in the absence of any protein factor [41,42]. This structural fine-tuning leads to their mutual functional regulation [42,43]. Whether this is the case for the mechanism involved in the 3′ genomic UTR-induced enhancement of WNV translation remains to be seen. Experiments to test this hypothesis are being planned at our laboratory. It is also unknown whether the 3′ UTR uses the same mechanism to potentiate cap-dependent and cap-independent translation initiation. Although other options cannot be ruled out, the fact that translation enhancement is reproducible in mosquito and different mammalian cell lines, and that it occurs in trans, argues for the direct involvement of the 3′ UTR structural elements. These might lead to the recruitment of factors present in all cell lines, or promote the structural reorganization of the 5′ end. The results obtained with the 3′ UTR variants lacking specific structural elements reinforce the latter hypothesis, and suggest the involvement of specific structural motifs. The elimination of the 5′DB element (dPK3) led to a reduction in the translation efficiency of m^7^G-WNV, both in Vero and mosquito cells (Figure 6), reproducing the effect observed with the heterologous DB1 deletion mutant of DTMUV [7]. Furthermore, the surprising enhancement observed for the SL-III deletion mutant in both cell lines, and to a lesser extent for the PK1 and/or its surrounding structural elements (SL-II and RCS3), may account for the fine regulation of 3′ UTR-mediated translation initiation—the outcome of the balance of opposing effects involving different 3′ UTR structural elements.

The above results suggest different scenarios via which the 3′ UTR achieves translation regulation, which may involve the recruitment of cellular factors by specific structural elements with opposite effects on translation. The active folding of SL-III, PK1, SL-II and RCS3 might be influenced by the surrounding elements, and tertiary interactions that influence the structure and function at the translation initiation site cannot be ruled out. The negative regulation of translation initiation observed for SL-III and PK1 (and/or SL-II and RCS3, which have been also deleted in the dPK1 construct) is reminiscent of that associated with the 5′BSL3.2 structural element at the 3′ end of the HCV genome [31,44]. In earlier work, we showed that 5′BSL3.2 negatively regulates HCV IRES-dependent translation. The molecular mechanism involves a direct RNA/RNA interaction (mediated by sequence complementarity) between 5′BSL3.2 and the universally conserved IIId subdomain of the HCV IRES. However, no sequence complementarity that might support direct RNA-RNA interaction has been detected between the SL-III domain and the 5′ end of WNV. It is nonetheless intriguing that members of different genera of the family *Flaviviridae* should conserve a negative regulation of the translation mechanism exerted via specific structural elements located at the 3′ end of the viral RNA genome. It may be that this reflects a general strategy of translation regulation for all members of the family.

The enhancement in translation initiation induced by the wild type 3′ UTR was similar in both the capped and uncapped context. However, greater translation efficiency was observed for all deletion variants when compared to the corresponding wild type construct in the cap-independent context (Figure 6). The reason for this is currently being examined at our laboratory.

In conclusion, the present results clearly show that the genomic 3′ UTR of flaviviruses is a translation enhancer. Its effect may be the result of balancing the participation in translation initiation of structural elements with different and even opposing functions. The 3′ UTR enhances both the cap-dependent and cap-independent initiation translation mechanisms. The 5′ end of WNV retains a cap-independent initiation of translation mechanism, as observed in other flaviviruses, the function of which is dependent on the presence of a flavivirus genomic 3′ UTR.

## 4. Materials and Methods

### 4.1. Transcription DNA Templates and RNA Constructs

The WNV genomic fragments used in this study were obtained by PCR amplification of the NY99-flamingo382–99 strain with the modifications described by Martín-Acebes and Saiz (GeneBank access AF196835) [45,46]. The coding sequence of the 5′ end of the WNV genome spanning nt 1–160 was obtained using a pair of primers that incorporated a *Kpn*I restriction enzyme site followed by the promoter sequence of the T7 RNA polymerase at the 5′ end of the amplification product, and a *Hind*III restriction enzyme site at its 3′ end. The primers used to amplify the coding sequence of the genomic 3′ UTR (nt 10,399 to 11,029) introduced an *Xba*I and a *Bam*HI site at the 5′ and 3′ ends of the amplification product, respectively. The plasmid pGLWNV for the WNV RNA construct was generated in three steps: first the 5′ PCR amplification product was cloned in the *Kpn*I-*Hind*III sites of the pGL3 Basic vector (Promega, Hercules, CA, USA). The 3′ amplification product was then introduced into the *Xba*I-*Bam*HI sites of the resulting plasmid. Finally, the luciferase AUG codon was removed by site-directed mutagenesis using the Phusion Site-Directed Mutagenesis Kit (Thermo Scientific™, Waltham, MA, USA). In the resulting pGLWNV plasmid, the FLuc coding sequences is in phase with the WNV AUG, and is flanked by the 5′ end and the 3′ UTR of the NY99-flamingo382–99 WNV strain. The WNV RNA construct was obtained by in vitro transcription from the *Bam*HI linearized pGLWNV plasmid. Plasmid linearization with *Xba*I yielded the template for the WNVΔ3′UTR construct.

DNA templates encoding the FLuc-WNV were obtained by PCR from the plasmid pGLWNV using primer T7pFLuc [31] and the antisense primer of the WNV 3′ UTR end. The plasmid template for the I3′WNV construct was obtained by replacing the *Xba*I-*Bam*HI fragment of the pGL-ICU plasmid [31] by the *Xba*I-*Bam*HI fragment encompassing the WNV 3′ UTR from plasmid pGLWNV. DNA templates encoding the chimeric WNV_3′DENV and WNV_3′YFV RNA constructs were obtained by replacing the 3′ UTR of plasmid pGLWNV by the 3′ UTR of DENV and YFV, respectively. DNA fragments encoding the 3′ UTR of DENV and YFV were purchased from Integrated DNA Technologies (Coralville, IA, USA). Plasmid DNA encoding the chimeric subgenomic RNA construct was obtained as follows. First, the WNV 3′ UTR coding sequence was deleted from the plasmid pGLWNV using the Phusion Site-Directed Mutagenesis Kit (Thermo Scientific) following the manufacturer’s instructions, and using an appropriate pair of primers that added *Bam*HI and *Sal*I restriction enzymes sites at the 3′ end of the FLuc coding sequence. The fragments encoding the 3′ UTRs of DENV and YFV were introduced by recombination between the *Bam*HI and *Sal*I sites using the In-Fusion^®^ HD Cloning Kit (Takara, Kusatsu, Japan) to yield plasmids pGLWNV-3′DENV and pGLWNV-3′YFV. *Sal*I digestion of the plasmids yielded DNA templates for the corresponding chimeric RNA constructs.

The DNA template for the dicistronic construct pRL-WNV was obtained by recombination using the In-Fusion^®^ HD Cloning Plus kit from Takara, following the manufacturer’s instructions. This construct bears the RLuc protein coding sequence followed by the 5′ UTR of the WNV fused to the FLuc protein coding sequence, in which the FLuc AUG start codon has been deleted. The 3′ UTR of the WNV genome is attached to this cassette at the latter’s 3′ end. To generate the DNA, the pRL-SV40 receptor plasmid (Promega) was digested with the endonucleases *Xba*I and *Bam*HI. The cassette bearing the 5′UTR-FLuc-3′UTR was then obtained by PCR from the pGLWNV DNA template. Primers were designed to include flanking sequences that would facilitate homologous recombination with the receptor plasmid, avoiding the recognition of the *Xba*I site. Thus, only one *Xba*I site at the 3′ end of the FLuc gene remains in the resulting DNA construct.

DNA templates pRL-WNV_del5′ and pRL-WNV_del5′_AUG_ were obtained from the parental DNA pRL-WNV by site-directed mutagenesis using appropriate primers and the Phusion Site-Directed Mutagenesis Kit (Thermo Scientific) following the manufacturer’s instructions. The pRL-WNV_del5′ plasmid codes for a deleted version of the 5′ UTR (comprising only nt 1–95) that lacks the starting AUG, whereas pRL-WNV_del5′_AUG_ bears nt1–98, thus incorporating this start codon sequence.

The pRL-WNV, pRL-WNV_del5′ and pRL-WNV_del5′_AUG_ plasmids were digested with the restriction enzyme *Bam*HI and in vitro transcribed from the T7 promoter sequence attached at the 5′ end of the *rluc* gene using T7 RNA polymerase, to yield the m^7^G-DIC, m^7^G-DIC_del5′ and the m^7^G-DIC_del5′_AUG_ dicistronic RNAs, respectively. Alternatively, the pRL-WNV DNA was digested with the endonuclease *Xba*I to generate a truncated version lacking the WNV 3′ UTR, named m^7^G-DIC_del3′UTR. These transcription reactions were performed in the presence of a cap analogue nucleotide (see below).

The DNA templates encoding the mutant RNA constructs dPK1, dPK2, dPK3 and dSLIII with deletions of different structural elements were generated by site-directed mutagenesis (using the Phusion Site-Directed Mutagenesis Kit) from the pGLWNV template plasmid using appropriate pairs of primers. The dPK1 construct lacked the structural elements involved in the formation of the PK1 structure (the deletion runs from nt 10,501 to 10,582); the dPK2 construct lacked the structural elements involved in the formation of the PK2 structure (the deletion runs from nt 10,667 to 10,734), and the dPK3 construct lacked the structural elements involved in the formation of the PK3 structure (the deletion runs from nt 10,761 to 10,827). The dSLIII construct lacked the entire SL-III element (nucleotides 10,599 to 10,655). The resulting DNA plasmids were digested with the *Bam*HI restriction enzyme to yield the DNA templates for the corresponding RNA constructs. The DNA template for the RNA-100 was obtained as previously described [22].

The primer sequences used in this work can be provided upon request.

All RNAs were synthesized using the HighYield T7 RNA Synthesis kit (Jena Bioscience, Thuringen, Germany) following the manufacturer’s instructions. Synthesis of the RNA RLuc and the addition of the 5′ cap was as previously described [47]. All transcripts were purified and quantified as previously described [41].

### 4.2. Cell Culture Conditions

African green monkey kidney cells (Vero) were maintained in Minimum Essential Medium (MEM) supplemented with 5% heat-inactivated foetal bovine serum (FBS; Gibco^®^ by LifeTechnologies™, Invitrogen, Waltham, MA, USA), 2 mM L-Glutamine (Sigma, St. Louis, MO, USA) and 1 mM sodium pyruvate (Sigma). Baby hamster kidney (BHK-21) cells were maintained in Glasgow Minimum Essential Medium (G-MEM) supplemented with 10% heat-inactivated foetal bovine serum (FBS), 0.1 mM tryptose phosphate and 10 mM HEPES buffer (Sigma). Human embryonic kidney cells (HEK-293T) and human cervical cancer cells (HeLa) were maintained in Dulbecco’s Modified Eagle Medium (DMEM-LG) supplemented with 10% heat-inactivated foetal bovine serum, 2 mM L-Glutamine and 0.1 mM non-essential amino acids (Gibco^®^ by LifeTechnologies™). Cells were incubated at 37 °C in a 5% CO_2_ atmosphere.

C6/36 cells of Aedes albopictus larvae were maintained in M3 Insect medium supplemented with 10% heat-inactivated foetal bovine serum, 2 mM L-Glutamine and 0.1 mM non-essential amino acids at 28 °C in a 5% CO_2_ atmosphere.

### 4.3. Transfection Assays

Cell transfections were essentially performed as previously described [47]. Briefly, 24 h before transfection, a specific number of cells (60 × 103 Vero cells, 90 × 103 BHK-21 cells, 110 × 103 HeLa cells, 200 × 103 HEK-293T cells, and 500 × 103 C6/36 cells) was seeded onto a 24-well plate to reach approximately 80% confluence. 1.5 µg of the RNA construct to be studied and 0.30 µg of the cap-RLuc were then mixed with 100 µL of Opti-MEM^®^ (Gibco^®^ by LifeTechnologies™) and 2 µL of transfection reagent (TransFectin™; Bio-Rad, Hercules, CA, USA) for well-to-well normalisation. This lipid–RNA complex was added to the cell culture.

### 4.4. Luciferase Measurement Assays

Translational efficiency was determined by measuring firefly and Renilla luciferase activities using the Dual-Luciferase Reporter Assay Kit (Vazyme, Nanjing, China) following the manufacturer’s instructions. All measurements were performed at 4 h post-transfection, and relative translation efficiency was calculated from the FLuc/RLuc ratio and then referred to that obtained by the reference RNA construct indicated in each assay.

## Data Availability

Primer sequences used in this work will be provided upon request.

## References

[1] Ray D., Shah A., Tilgner M., Guo Y., Zhao Y., Dong H., Deas T.S., Zhou Y., Li H., Shi P.Y. (2006). West Nile virus 5′-cap structure is formed by sequential guanine N-7 and ribose 2′-O methylations by nonstructural protein 5. J. Virol..

[2] Zhou Y., Ray D., Zhao Y., Dong H., Ren S., Li Z., Guo Y., Bernard K.A., Shi P.Y., Li H. (2007). Structure and function of flavivirus NS5 methyltransferase. J. Virol..

[3] Aitken C.E., Lorsch J.R. (2012). A mechanistic overview of translation initiation in eukaryotes. Nat. Struct. Mol. Biol..

[4] Paranjape S.M., Harris E. (2010). Control of Dengue virus translation and replication. Curr. Top. Microbiol. Immunol..

[5] Edgil D., Polacek C., Harris E. (2006). Dengue virus utilizes a novel strategy for translation initiation when cap-dependent translation is inhibited. J. Virol..

[6] Song Y., Mugavero J., Stauft C.B., Wimmer E. (2019). Dengue and Zika virus 5′ untranslated regions harbor internal ribosomal entry site functions. mBio.

[7] Wang T., Merits A., Wu Y., Wang M., Jia R., Zhu D., Liu M., Zhao X., Yang Q., Wu Y. (2020). Cis-acting sequences and secondary structures in untranslated regions of duck Tembusu virus RNA are important for cap-independent translation and viral proliferation. J. Virol..

[8] Fernández-Sanlés A., Ríos-Marco P., Romero-López C., Berzal-Herranz A. (2017). Functional information stored in the conserved structural RNA domains of flavivirus genomes. Front. Microbiol..

[9] Ng W.C., Soto-Acosta R., Bradrick S.S., Garcia-Blanco M.A., Ooi E.E. (2017). The 5′ and 3′ untranslated regions of the flaviviral genome. Viruses.

[10] Ramos-Lorente S., Romero-Lopez C., Berzal-Herranz A. (2021). Information encoded by the flavivirus genomes beyond the nucleotide sequence. Int. J. Mol. Sci..

[11] Wengler G. (1981). Terminal sequences of the genome and replicative-from RNA of the flavivirus West Nile virus: Absence of poly(A) and possible role in RNA replication. Virology.

[12] Brinton M.A., Fernandez A.V., Dispoto J.H. (1986). The 3′-nucleotides of flavivirus genomic RNA form a conserved secondary structure. Virology.

[13] Li W., Brinton M.A. (2001). The 3′ stem loop of the West Nile virus genomic RNA can suppress translation of chimeric mRNAs. Virology.

[14] Wei Y., Qin C., Jiang T., Li X., Zhao H., Liu Z., Deng Y., Liu R., Chen S., Yu M. (2009). Translational regulation by the 3′ untranslated region of the Dengue type 2 virus genome. Am. J. Trop. Med. Hyg..

[15] Manzano M., Reichert E.D., Polo S., Falgout B., Kasprzak W., Shapiro B.A., Padmanabhan R. (2011). Identification of cis-acting elements in the 3′-untranslated region of the Dengue virus type 2 RNA that modulate translation and replication. J. Biol. Chem..

[16] Guo L., Allen E., Miller W.A. (2000). Structure and function of a cap-independent translation element that functions in either the 3′ or the 5′ untranslated region. RNA.

[17] Shatsky I.N., Dmitriev S.E., Terenin I.M., Andreev D.E. (2010). Cap- and IRES-independent scanning mechanism of translation initiation as an alternative to the concept of cellular IRESs. Mol. Cells.

[18] Terenin I.M., Andreev D.E., Dmitriev S.E., Shatsky I.N. (2013). A novel mechanism of eukaryotic translation initiation that is neither m7G-cap-, nor IRES-dependent. Nucleic Acids Res..

[19] Nicholson B.L., White K.A. (2011). 3′ cap-independent translation enhancers of positive-strand RNA plant viruses. Curr. Opin. Virol..

[20] Simon A.E., Miller W.A. (2013). 3′ cap-independent translation enhancers of plant viruses. Annu. Rev. Microbiol..

[21] Clyde K., Harris E. (2006). RNA secondary structure in the coding region of Dengue virus type 2 directs translation start codon selection and is required for viral replication. J. Virol..

[22] Romero-López C., Ríos-Marco P., Berzal-Herranz B., Berzal-Herranz A. (2018). The HCV genome domains 5BSL3.1 and 5BSL3.3 act as managers of translation. Sci. Rep..

[23] Bidet K., Garcia-Blanco M.A. (2014). Flaviviral RNAs: Weapons and targets in the war between virus and host. Biochem. J..

[24] Bidet K., Garcia-Blanco M.A. (2018). Flaviviral RNA structures and their role in replication and immunity. Adv. Exp. Med. Biol..

[25] Jan E., Sarnow P. (2002). Factorless ribosome assembly on the internal ribosome entry site of cricket paralysis virus. J. Mol. Biol..

[26] Gross L., Vicens Q., Einhorn E., Noireterre A., Schaeffer L., Kuhn L., Imler J.L., Eriani G., Meignin C., Martin F. (2017). The IRES5′UTR of the dicistrovirus cricket paralysis virus is a type III IRES containing an essential pseudoknot structure. Nucleic Acids Res..

[27] Kirby M.P., Stevenson C., Worrall L.J., Chen Y., Young C., Youm J., Strynadka N.C.J., Allan D.W., Jan E. (2022). The hinge region of the israeli acute paralysis virus internal ribosome entry site directs ribosomal positioning, translational activity, and virus infection. J. Virol..

[28] Yang Y., Wang Z. (2019). IRES-mediated cap-independent translation, a path leading to hidden proteome. J. Mol. Cell Biol..

[29] Gokhale N.S., McIntyre A.B.R., Mattocks M.D., Holley C.L., Lazear H.M., Mason C.E., Horner S.M. (2020). Altered m(6)A modification of specific cellular transcripts affects flaviviridae infection. Mol. Cell.

[30] Bung C., Bochkaeva Z., Terenin I., Zinovkin R., Shatsky I.N., Niepmann M. (2010). Influence of the hepatitis C virus 3′-untranslated region on IRES-dependent and cap-dependent translation initiation. FEBS Lett..

[31] Romero-López C., Berzal-Herranz A. (2012). The functional rna domain 5BSL3.2 within the NS5b coding sequence influences hepatitis C virus IRES-mediated translation. Cell. Mol. Life Sci..

[32] Bai Y., Zhou K., Doudna J.A. (2013). Hepatitis C virus 3′UTR regulates viral translation through direct interactions with the host translation machinery. Nucleic Acids Res..

[33] Brinton M.A. (2014). Replication cycle and molecular biology of the West Nile virus. Viruses.

[34] Khromykh A.A., Meka H., Guyatt K.J., Westaway E.G. (2001). Essential role of cyclization sequences in flavivirus RNA replication. J. Virol..

[35] Lo M.K., Tilgner M., Bernard K.A., Shi P.Y. (2003). Functional analysis of mosquito-borne flavivirus conserved sequence elements within 3′ untranslated region of West Nile virus by use of a reporting replicon that differentiates between viral translation and RNA replication. J. Virol..

[36] Alvarez D.E., Lodeiro M.F., Luduena S.J., Pietrasanta L.I., Gamarnik A.V. (2005). Long-range RNA-RNA interactions circularize the Dengue virus genome. J. Virol..

[37] Zhang B., Dong H., Stein D.A., Iversen P.L., Shi P.Y. (2008). West Nile virus genome cyclization and RNA replication require two pairs of long-distance RNA interactions. Virology.

[38] Friebe P., Harris E. (2010). Interplay of RNA elements in the Dengue virus 5′ and 3′ ends required for viral RNA replication. J. Virol..

[39] Li X.D., Deng C.L., Yuan Z.M., Ye H.Q., Zhang B. (2020). Different degrees of 5′-to-3′ DAR interactions modulate Zika virus genome cyclization and host-specific replication. J. Virol..

[40] Sanford T.J., Mears H.V., Fajardo T., Locker N., Sweeney T.R. (2019). Circularization of flavivirus genomic RNA inhibits de novo translation initiation. Nucleic Acids Res..

[41] Romero-López C., Barroso-Deljesus A., García-Sacristán A., Briones C., Berzal-Herranz A. (2012). The folding of the hepatitis C virus internal ribosome entry site depends on the 3′-end of the viral genome. Nucleic Acids Res..

[42] Romero-López C., Barroso-Deljesus A., García-Sacristán A., Briones C., Berzal-Herranz A. (2014). End-to-end crosstalk within the hepatitis C virus genome mediates the conformational switch of the 3′X-tail region. Nucleic Acids Res..

[43] Romero-López C., Barroso-delJesus A., Berzal-Herranz A. (2017). The chaperone-like activity of the hepatitis C virus IRES and CRE elements regulates genome dimerization. Sci. Rep..

[44] Romero-López C., Berzal-Herranz A. (2009). A long-range RNA-RNA interaction between the 5′ and 3′ ends of the HCV genome. RNA.

[45] Lanciotti R.S., Roehrig J.T., Deubel V., Smith J., Parker M., Steele K., Crise B., Volpe K.E., Crabtree M.B., Scherret J.H. (1999). Origin of the West Nile virus responsible for an outbreak of encephalitis in the northeastern United States. Science.

[46] Martin-Acebes M.A., Saiz J.C. (2011). A West Nile virus mutant with increased resistance to acid-induced inactivation. J. Gen. Virol..

[47] Romero-López C., Díaz-González R., Berzal-Herranz A. (2007). Inhibition of hepatitis C virus internal ribosome entry site-mediated translation by an RNA targeting the conserved IIIf domain. Cell. Mol. Life Sci..

